# Which Factors Affect the Success or Failure of Eradication Campaigns against Alien Species?

**DOI:** 10.1371/journal.pone.0048157

**Published:** 2012-10-26

**Authors:** Therese Pluess, Vojtěch Jarošík, Petr Pyšek, Ray Cannon, Jan Pergl, Annemarie Breukers, Sven Bacher

**Affiliations:** 1 Department of Biology, Ecology & Evolution Unit, University of Fribourg, Fribourg, Switzerland; 2 Department of Ecology, Charles University in Prague, Faculty of Science, Praha, Czech Republic; 3 Institute of Botany, Academy of Sciences of the Czech Republic, Průhonice, Czech Republic; 4 The Food and Environment Research Agency, Sand Hutton, York, United Kingdom; 5 LEI, part of Wageningen UR, Wageningen, The Netherlands; University of Utah, United States of America

## Abstract

Although issues related to the management of invasive alien species are receiving increasing attention, little is known about which factors affect the likelihood of success of management measures. We applied two data mining techniques, classification trees and boosted trees, to identify factors that relate to the success of management campaigns aimed at eradicating invasive alien invertebrates, plants and plant pathogens. We assembled a dataset of 173 different eradication campaigns against 94 species worldwide, about a half of which (50.9%) were successful. Eradications in man-made habitats, greenhouses in particular, were more likely to succeed than those in (semi-)natural habitats. In man-made habitats the probability of success was generally high in Australasia, while in Europe and the Americas it was higher for local infestations that are easier to deal with, and for international campaigns that are likely to profit from cross-border cooperation. In (semi-) natural habitats, eradication campaigns were more likely to succeed for plants introduced as an ornamental and escaped from cultivation prior to invasion. Averaging out all other factors in boosted trees, pathogens, bacteria and viruses were most, and fungi the least likely to be eradicated; for plants and invertebrates the probability was intermediate. Our analysis indicates that initiating the campaign before the extent of infestation reaches the critical threshold, starting to eradicate within the first four years since the problem has been noticed, paying special attention to species introduced by the cultivation pathway, and applying sanitary measures can substantially increase the probability of eradication success. Our investigations also revealed that information on socioeconomic factors, which are often considered to be crucial for eradication success, is rarely available, and thus their relative importance cannot be evaluated. Future campaigns should carefully document socioeconomic factors to enable tests of their importance.

## Introduction

The focus of much recent research on biological invasions has shifted away from theoretical considerations towards more practical issues, particularly concerning the ecological and economic impacts of invasive species [1]–[7]. There is also an urgent need to understand how best to manage alien invasive species, and if necessary, how to eradicate them completely from an invaded area [8]–[10]. Yet, almost nothing is known about how environmental settings affect the outcome of such management actions since the issue has not been rigorously evaluated. If measures to prevent the introduction of an invasive species fail, eradication is regularly considered as an option, in order to avoid impacts the invasive species might otherwise cause. Eradication aims at eliminating an organism from an area or management unit [10], [11].

Recently, there has been a renewed interest in the eradication of invasive species, following a period when the prevailing view was that eradication was very seldom achievable [10], [12]. Some eradication campaigns have been successful, especially when initiated at an early stage of the invasion [13]–[16]. However, it is sometimes difficult to respond to the incursion appropriately, i.e. in terms of choosing the most effective eradication strategy, since the outcome of such efforts depends on many different factors, related to both the invading species itself and the environmental settings of the infestation or outbreak. Thus, when responsible authorities are confronted with the outbreak of a given invasive species, knowing which are the key factors to focus on in terms of achieving an eradication success, would be very valuable.

In the last two decades, a number of reviews of eradication attempts have compared the outcome for various taxonomic groups, either based on descriptive case studies [12], [15], [17]–[20] or by assessing taxonomic groups separately: such as plants [14], [21], [22], mammals [23], [24], moths [25], invertebrates [26], or plant pathogens [27]. Simberloff [12] argued that eradication is feasible more often than is reflected in the current literature. These studies suggest, but do not quantitatively test, that the following key factors affect the success or failure of eradication campaigns: reaction time, the extent of outbreak, the knowledge of the invading species’ biology (which is also associated with the preparedness of authorities to react), and whether the campaign was on an island or the geographical mainland [12], [23]. However, in a previous paper, we rigorously tested the effects of these factors on the outcome of 136 systematically assembled eradication campaigns against invasive alien invertebrates, plants and pathogens by using generalized linear models, and found that the only factor which was significantly related to eradication success was the extent of the infestation [28].

To obtain a deeper insight into factors relating to the success or failure of eradication campaigns, we here apply two techniques of predictive data mining, classification [29], [30] and boosted trees [31], [32], on a broadened assemblage of 173 eradication campaigns. The use of this larger dataset was enabled by the fact that data mining techniques can handle data gaps by calculating surrogate variables to replace missing values; for the generalized linear model, all cases with missing values had to be discarded. The data mining techniques can also reveal additional factors relevant for eradication success that may have been overlooked when tested by classical statistical approaches [7], [33]. Unlike the classical linear methods, the data mining techniques enable predictions to be made from the data and to identify the most important predictors by screening a large number of candidate variables without requiring the user to make any assumptions about the form of the relationships between the explanatory and the response variables, and without *a priori* formulated hypotheses [34]. These techniques are also more flexible than traditional statistical analyses because they can reveal structures in the dataset that are other than linear, and solve complex interactions (e.g. some factors being only relevant for certain taxa or under certain environmental conditions). Classification trees [29], [30] provide easily understandable graphical presentations of the relationships between predictors and the outcome of eradication campaigns, and enable one to construct trees with potentially different structure by artificially placing some factors at the top of a tree [35]. Boosted trees [31], [32], which can be seen as an extension of classification trees by fitting many sub-trees to parts of the dataset and then combining the predictions from all trees, are a convenient tool because of their ability to graphically characterize relationships between the individual predictors and probabilities of prediction success [32], [36].

We divided the success factors into three categories originally suggested for evaluating the establishment success of exotic birds [37], [38]: species-specific, location-specific and event-specific success factors. By dividing factors into these categories, we attempt to disentangle whether a campaign was successful as a result of the (i) biological traits of the organism (species-specific), (ii) environmental settings at the infested site (location-specific), (iii) features of the current outbreak situation (event-specific), or (iv) interactions between these factors. Whilst the properties of the species or the location of an outbreak cannot be changed by the managing authorities – because they are intrinsic characteristics related to the given species or location – most event-specific factors can potentially be addressed by a proper planning and management strategy. For instance, the appropriate choice of management measures, quick reaction time, high level of preparedness of an authority to react to an outbreak, good stakeholder cooperation and public support are widely believed to be crucial for eradication success e.g. [12]. Therefore, we also (v) explicitly test how the manageable event-specific factors affect the eradication success, to provide managers with information that can be directly applied to choosing the most appropriate strategy in a specific situation.

## Materials and Methods

### Data Collection

We collated information on eradication campaigns aimed at eliminating an invasive species from an area and directed against invertebrate plant pests, plant pathogens (viruses/viroids, bacteria and fungi) and weeds. Campaigns from the entire world, started between 1914 and 2009, were considered.

The scientific and grey literature were searched for information on eradication programmes, including those that had been published in scientific journals and books, eradication or other technical reports, pest alerts and press releases from National Plant Protection Organizations (NPPOs). Information about eradication campaigns is often difficult to obtain [12], so national and regional plant protection organizations were another important source of information for this study. Pest managers from NPPOs (see Acknowledgements) were contacted and asked to provide examples and detailed information about eradication campaigns from their countries. The list of all cases is given in Table S1.

### Explanatory Variables

Factors which were assumed to affect whether or not eradication campaigns were successful, were identified, both on the basis of discussions with experienced pest managers and by reviewing the literature [12], [18], [20]. Twenty-nine explanatory variables, with on average 13% of missing values, were divided into three groups (Table 1), and used in analyses:

- Species-specific factors included the taxonomic affiliation; how easily the organism can be identified; and the economic sectors it affected.
- Location-specific factors included information about the invaded habitat type (classified according to the European Nature Information System EUNIS [39]; http://eunis.eea.europa.eu/habitats.jsp), distinguishing between man-made habitats (EUNIS category I; regularly or recently cultivated agricultural, horticultural and domestic habitats or EUNIS category J; constructed, industrial and other artificial habitats) and (semi-)natural habitats (EUNIS categories A to H). Other factors described the insularity and the accessibility of the infestation; whether it concerned an indoor or an outdoor system; and the world region in which it occurred.
- Event-specific factors related to the extent of the invasion were the percentage of infested habitat; pest distribution; management measures and their availability; the level of biological knowledge available to the authorities; detection mode; reaction time; coordination; introduction rate; and pathways of entry.

**Table 1:** Description of 29 potential success factors of 173 eradications against invertebrate plant pests, plant pathogens (viruses/viroids, bacteria and fungi) and weeds, used in data mining analyses.

Factor	Description
Outcome	Success: the eradication campaign was reported to be successful, which was confirmed by surveys over a time period relevant for the life-cycle of the organism, the campaign stopped, N = 88
	Failure: all other campaigns, N = 85
Weight	1/(number of campaigns for this species); number between 1 and 0.01667
**Species-specific factors**	
Kingdom	Taxonomic kingdom: Virus-like organisms, Bacteria, Fungi, Animalia (represented by invertebrates), Plantae
Identification method	Methods needed for the identification of the organism:
	Eye: identifiable with naked eye, N = 98
	Microscope: microscope and literature needed for identification, N = 35
	Molecular: more complex tools/molecular tools needed to identify species, N = 38
	NA: 2
Agricultural problem	Yes: organism considered an agricultural problem (all annual and perennial outdoor and indoor agricultural and horticultural crops) N = 82
	No: organism not considered an agricultural problem, N = 66
	NA: 25
Forestry problem	Yes: organism considered a problem in (managed) forests or tree plantations, N = 38
	No: organism not considered a forestry problem, N = 110
	NA: 25
**Location specific factors**	
Man-made habitat	Yes: campaign in EUNIS habitats* I and/or J, N = 114
	No: campaign did not encompass EUNIS habitats I/J, N = 59
Insularity	eradication on an island, N = 53
	eradication on the mainland, N = 120
Accessibility	Yes: access to private properties problematic, remote areas concerned, N = 19
	Sometimes: some difficulties to access the target area, N = 12
	No: no difficulties to access the target area, N = 115
	NA: 27
Indoor or outdoor habitat	Indoor: campaign in protected cultures (greenhouses), N = 26
	Outdoor: campaign in outdoor habitats, N = 134
	Both: campaign in both indoor and outdoor habitats, N = 13
World region	Americas: North and South America, including Pacific islands east of the international dateline, N = 53
	Australasia: including islands of the Pacific west of the international dateline, N = 36
	Europe, N = 84
**Event-specific factors**	
*General aspects of the outbreak situation*	
Spatial extent of outbreak	Local: One rather small, isolated outbreak focus, N = 66
	Regional: A larger area, but never the entire country, was affected including more than one and up to ten outbreak foci, N = 42
	National: campaign in entire country or on an entire island, usually including more than ten outbreak foci. For campaigns in the United States, Canada and Australia, states or provinces were classified as “national”, N = 50
	International: campaign involves several countries (or states or provinces in the case of the USA, Canada or Australia), number of outbreak foci is irrelevant, N = 11
	NA: 4
Area infested [ha]	Size of infested area in hectares, as reported. Often, the treated area was given, as exact extension was not known at onset of measures. Treated area is taken, if no other information was available. If area increases over time, the largest size was taken.
	NA: 73
Proportion infested [%]	Proportion of suitable habitat infested at the onset of the measures
	NA: 107
Pest distribution	Patchy: several, reproductively isolated populations, N = 139
	Continuous: one continuous population, N = 19
	NA: 15
*Measures & preparedness*	
Biological control	Yes: biocontrol measures, including Sterile Insect Techniques, N = 27
	No: no use of biocontrol measures, N = 145
	NA: 1
Chemical control	Yes: chemical measures; include spraying of *Bacillus thuringiensis* and/or use of pheromone traps, male annihilation, N = 122
	No: no use of chemical control measures, N = 52
	NA: 1
Cultural control	Yes: changed crop rotation, planting of resistant hosts, N = 30
	No: no use of cultural control measures, N = 142
	NA: 1
Physical control	Yes: uprooting, burning, chipping and other disposal methods of plant material, N = 123
	No: no use of physical control measures, N = 49
	NA: 1
Sanitary control	Yes: Movement of possibly infested plant material or equipment prohibited, N = 54
	No: no use of sanitary measures, N = 118
	NA: 1
Measures available	Yes: control measures were available at moment of outbreak, N = 98
	Some: some measures were available, others not (yet), N = 7
	No: measures had to be developed during campaign, N = 9
	NA: 59
Knowledge and preparedness	None: Information about the species and possible management measures were collected and evaluated after the incursion, N = 16
	Low: Pest alerts, pest notices or similar information were available when the pest was detected, N = 58
	Medium: A Pest Risk Analysis for the species or a generic contingency plan was available when the pest was detected or the pest was well known and control experience existed, N = 27
	High: A contingency plan against the species was available when the pest was detected, or a precise plan to eradicate the pest was mentioned, N = 54
	NA: 18
Official detection	Yes: infestation was detected during an official survey or inspection by plant protection authorities, N = 82
	No: infestation was not detected during a survey of authorities, N = 43
	NA: 48
Reaction time	The time elapsing between the arrival (or detection) of the organism and the start of the eradication campaign, counted in months
	NA: 17
Coordination	Self-declared or assumed level of coordination between involved parties
	None: N = 1
	Existing: N = 27
	Well functioning: N = 120
	NA: 25
*Introduction & pathways*	
Rate of introduction	Low: only introduced once, N = 8
	Medium: introduced once in ten years or less, N = 21
	High: introduced between once per year or once in nine years, N = 21
	Very high: introduced several times a year, N = 47
	NA: 76
Pathway**: Contaminant	Yes: introduction as contaminant of goods (e.g. plant material), N = 97
	No: introduction not as contaminant, N = 48
	NA: 28
Pathway: Corridor	Yes: introduction via a corridor; introduced via transport infrastructure, N = 13
	No: introduction not via a corridor, N = 132
	NA: 28
Pathway: Escaped	Yes: introduction by escaping from cultivation, N = 7
	No: introduction not by escaping from cultivation, N = 139
	NA: 27
Pathway: Stowaway	Yes: introduction as hitchhiker; e.g. with tyres, luggage, ballast water, no specific commodity, N = 24
	No: introduction not as hitchhiker, N = 121
	NA: 28
Pathway: Unaided	Yes: Introduction occurred unaided, as natural spread from an already infested area, N = 33
	No: introduction not via natural spread, N = 113
	NA = 27

NA  =  information not available.

* European Nature Information System habitat classification http://eunis.eea.europa.eu/habitats.jsp.

** pathways were defined according to [44].

Socioeconomic factors that are often mentioned by experts to be important for eradication success (summarized in [12]) are effort (in terms of money and manpower) and dedication to execute the measures (e.g. motivation of project leaders and workers, level of acceptance by the community etc.). We tried to get information on these, but it is rarely published or even internally recorded. Even rough estimates of costs, which are arguably the most straightforward information to get, were available only for less than half of the cases and these estimates relied on peoples’ opinions more often than on actual bookkeeping. We considered these data as not rigorous enough and did not include them in the analyses. This information gap points to the recommendation that socioeconomic data should be standardly recorded by managers responsible for eradication campaigns.

### Response Variable

The binary dependent variable was whether an eradication campaign was successful or had failed. Campaigns were considered to be successful if the target organism was officially declared as having been eradicated by the responsible authorities; otherwise it was treated as a failure. Exactly when a species can be declared as being definitely eradicated (i.e. how long after the management intervention) depends on the species itself, and the situation in which it occurred, and must take into account factors such as seed-bank longevity (at least for plants). Ideally, eradication success should be stated in terms of confidence limits that the species is not present [10]. However, as this is rarely the case, our dataset – including recent and still on-going eradication campaigns – might potentially be biased towards failures because campaigns still on-going as of December 2009 (the cut-off date for data collection) were considered as a failure, since successful eradication had not by then been declared. It could of course be achieved subsequent to this date in some cases.

### Statistical Analyses

#### Predictive mining

The explanatory variables consisted of the 29 potential success factors and the binary response variable was the outcome of the campaign (success or failure). The mining was done using binary recursive partitioning, with a best split made based on the Gini impurity measure [40], [41]. The process is binary because parent nodes are always split into exactly two child nodes by asking questions that have a “yes” (“success” in our case) or “no” (“failure”) answer, and it is recursive because the process is repeated by treating each child node as a new parent until all the data are exhausted. The analyses were conducted with balanced class weights [35], assuring that the binary success/failure status was treated as equally important for the purpose of achieving classification accuracy. Ten-fold cross-validations were used to obtain final models with the smallest cross-validated errors for interpretation. This cross-validation involves splitting the data into a number of smaller samples with similar distributions of the response variable. Models are then generated, excluding the data from each subsample in turn. For each model, the error rate was estimated from the subsample excluded in generating it and the cross-validated error for the overall model was then calculated e.g. [29], [40].

The quality of the final models was expressed as (a) their overall misclassification rates compared to null models with random assignment to the success/failure status having expected 50% misclassification rates [30] and (b) the overall number of misclassified cases for success and failure [40]. These values were expressed based on learning samples, i.e. the samples not used to build the models for assessment of cross-validation errors [30]. Following Bourg et al. [42], we also evaluated (c) specificity, i.e. the ability of the models to predict that a particular case does not result in a successful eradication when it did not, and (d) sensitivity, i.e. the ability of the models to predict that a particular case does result in a successful eradication when it did. These values were based both on the learning samples and the cross-validated samples, expressing the best estimates of the misclassifications that would occur if the models were to be applied to new data, assuming that the new data were drawn from the same distribution as the learning data [40].

Outcomes of campaigns against the same species can be expected to be correlated and should thus not be treated as independent. Hence, to avoid pseudo-replications because some species were eradicated several times in independent campaigns, cases from each species were weighted by the reciprocal number of campaigns against this particular species [40]. Missing cases were treated by back-up rules based on surrogates of each split. These surrogates describe splitting rules that closely mimic the action of the missing primary splitters [29], [40]. To prevent variables with missing values from having an advantage as splitters, the predictor variables were penalized according to the proportion of their missing values [40].

Calculations of classification trees were made in the commercial statistical software CART Pro^©^ v.6 [29], [35], [40] and calculations of boosted trees in the commercial statistical software Predictive Mining Suite^©^ (Salford Systems).

#### Classification trees

Classification trees were first constructed without any predetermined structure. The analyses were then repeated with event-specific factors, which can be potentially addressed by a proper planning and management strategy (see Table 1), placed at the top of the trees as the first two splits, and the species- and location-specific factors, which cannot be changed by the managing authorities, further at the bottom of the trees. This division was done because distinguishing the predictors over which the manager has no control from those that can be controlled to some degree [35] can provide useful information for designing the appropriate eradication strategy.

To determine optimal classification trees, a sequence of nested trees of decreasing size was constructed and their re-substitution relative errors were estimated. These estimates were then plotted against tree sizes, and the optimal trees were chosen based on minimum cross-validated errors [40]. Following De’ath & Fabricius [30], a series of 50 cross-validations was run for each sequence of nested trees. For classification trees constructed without predetermined structure, the series of 50 cross-validations were repeated for the smallest terminal node sizes 1, 5, 10, 15 and 20 cases, with a default minimum size of splitting nodes being twice the size of the terminal nodes. The smallest error was revealed for five cases, and this minimal size of terminal nodes was also used for classification trees with event-specific factors at the top of the hierarchy, to reach comparable results throughout both analyses.

Optimal trees with the overall smallest error were represented graphically, with the root standing for the undivided dataset at the top, and the terminal nodes, describing the most homogeneous groups of data points, at the bottom of the hierarchy. The quality of each split was expressed by its improvement value, corresponding to the overall misclassification rate at the node, with high scores of improvement values corresponding to splits of high quality. In graphical representations, the vertical depth of each node was expressed as proportional to its improvement value. Vertical depth of each node thus represented a value similar to explained variance in a linear model.

#### Boosted trees

From residual-like measures of previous trees, 500 five-node trees, i.e. trees with a number of nodes approximately equal to the square root of the number of predictor variables [43], were sequentially built by using a 0.01 learning rate [31], [32]. At each iteration, a tree was built from a 50% random subsample of the data, with the minimum number of training observations in the terminal nodes being three. The optimal tree, having the smallest cross-validated mean absolute error, was then chosen for interpretation.

Using the optimal tree, positive and negative probabilities for individual predictors, previously tested in generalized linear models as *a priori* factors for successful eradication campaigns [28], were visualized as partial dependence plots [32], [36]. These plots show effects of these factors, namely (i) taxonomic Kingdom (Animalia, Bacteria, Fungi, Plantae, Viruses), (ii) biogeographic region (Europe, Americas, Australasia), (iii) reaction time between the arrival/detection of the organism and the start of the eradication campaign, (iv) the spatial extent of the pest outbreak, (v) the level of biological knowledge and preparedness, and (vi) insularity, as net effects, i.e. averaging out the effects of the other predictors included in the optimal tree.

## Results

**Figure 1:**
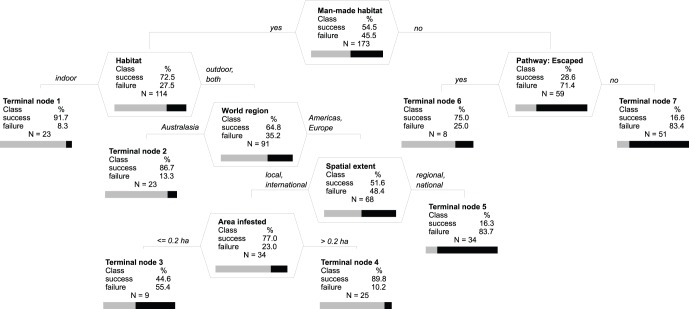
Optimal classification tree for factors relating to success and failure of 173 eradication campaigns against invertebrate plant pests, plant pathogens (viruses/viroids, bacteria and fungi) and weeds in a model without any predetermined structure. Splitting nodes (polygonal tables with splitting variable name) and terminal nodes (with a split criterion above each) show a table with columns for the outcome (success/failure) and % of weighted cases for each outcome, total number of unweighted cases (N), and graphical representation of the percentage of success (grey) and failure (black) weighted cases (horizontal bar). Vertical depth of each node is proportional to its improvement value that corresponds to explained variance at the node. Overall misclassification rate of the optimal tree is 15.8% compared to 50% for the null model, with 16.7% misclassified success and 14.8% failure cases. Sensitivity (true positive rate, defined as the ability of the model to predict that a case is eradicated when it actually is) is 83.3 and specificity (true negative rate, defined as the ability of the model to predict that a case is not eradicated when it is not) 85.2% for learning samples, i.e. the samples not used to build the models for assessment of cross-validation errors, and 77.1 and 69.0%, respectively, for cross-validated samples, i.e. the best estimates that would occur if the models were to be applied to new data, assuming that the new data were drawn from the same distribution as the learning data.

**Figure 2:**
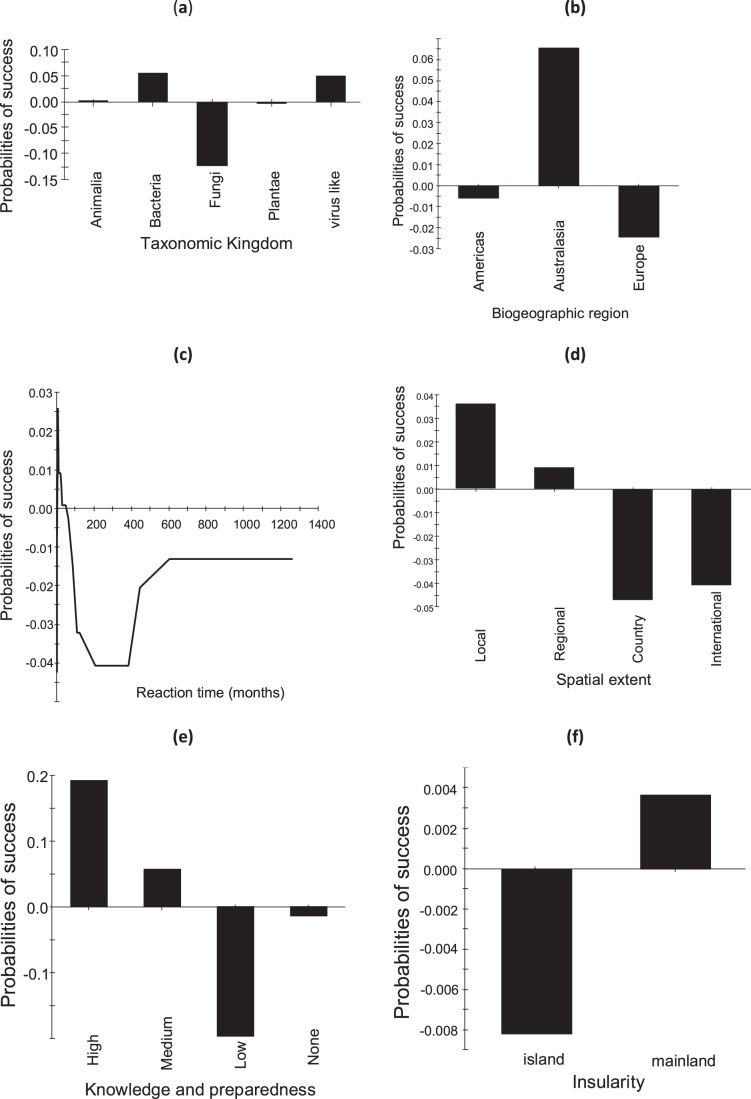
Partial dependence plots based on the optimal boosted tree for (a) taxonomic Kingdoms, (b) biogeographic regions, (c) the reaction time between the arrival/detection of the organism and the start of the eradication campaign, (d) the spatial extent of the pest outbreak, (e) the level of biological knowledge and preparedness, and (f) insularity. The plots show probabilities of success of an eradication campaign for these predictors as net effects, i.e. averaging out the effects of all the other predictors included in the optimal boosted tree. The optimal boosted tree has overall misclassification rate 5.2% with 3.0% misclassified success and 8.0% failure cases. Sensitivity and specificity are respectively 97.0 and 92.0% for learning, and 82.2 and 68.1% for cross-validated samples. See Table 1 for detail description of the predictors and Fig. 1 for detail explanation of misclassification rates, sensitivity and specificity.

**Figure 3:**
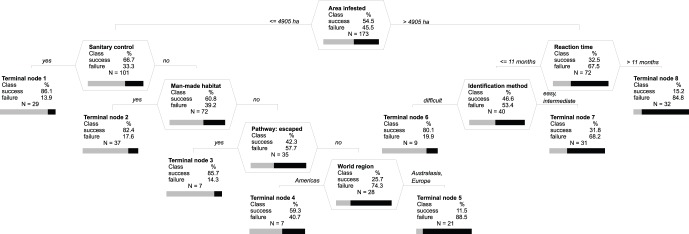
Optimal classification tree with event-specific factors placed at the top of the tree. Otherwise as in Fig. 1. Overall misclassification rate of the optimal tree is 18.0% with 15.3% misclassified success and 23.4% failure cases. Sensitivity and specificity are respectively 84.7 and 76.6% for learning, and 66.7 and 64.9% for cross-validated samples. Detail explanation of misclassification rates, sensitivity and specificity is in Fig. 1.

Of the 173 eradication campaigns which we examined, 91 were targeted against invertebrates (41 spp.), 55 against pathogens (26 spp. or subspp. of virus-like organisms, bacteria and fungi) and 27 against plants (27 spp.). In total, 88 (50.9%) campaigns were evaluated as having been successful, whilst 85 (49.1%) were unsuccessful. Model predictions of successes and failures were generally highly reliable, with low misclassification rates and a high ability to predict that a species was not eradicated when it was not (high specificity) and that a species was eradicated when it actually was (high sensitivity), including cross-validated samples (Figs 1, 2, 3). The cross-validated results suggest that specificity and sensitivity will remain high even if the actual models would be applied to new data.

Eradication campaigns were more successful in man-made habitats than in (semi)-natural habitats, especially if carried out indoor, e.g. in greenhouses where 91.7% of campaigns resulted in eradication (Fig. 1, Terminal Node 1). Of those in man-made habitats (either only outdoor or a mix of indoor/outdoor campaigns), campaigns in Australasia were successful in 86.7% of attempts (Terminal Node 2) while in the Americas and Europe success depended on the spatial extent of the infestation. In the latter two world areas, eradications were more successful at local or international scales than at regional or national scales (Terminal Node 5). At the local and international scale the success further depended on infested area, which however contributed to the overall quality of the model by just less than 6%. In (semi-)natural habitats, eradication campaigns were more likely to succeed if the targeted invading species was introduced for cultivation from where it escaped (Terminal Node 6).

Averaging out the effects of all the other predictors included in the optimal boosted tree, partial dependence plots (Fig. 2) indicated that the probability of success was lowest for eradication campaigns against fungi, highest for those against other microorganisms, and intermediate against invertebrates and plants (Fig. 2a). Campaigns in Australasia had the highest, and those in Europe the lowest probability of success (Fig. 2b). The effect of early detection was important. A period of approximately four years between the arrival or detection of the organism and the start of the eradication campaign was found to be a threshold, below or above which the success was likely or not, respectively. In other words, eradication campaigns should commence within four years of detection, on average, to achieve success. For campaigns which commenced 50 or more years after the start of the invasion, there was no relationship between the probability of success and the year of invasion (Fig. 2c). Large spatial extent (Fig. 2d), and low or no preparedness (Fig. 2e), were other factors decreasing the probability of success. Surprisingly, the probability of the net effect of success was lower on islands than on the mainland (Fig. 2f).

Artificially placing event-specific factors, over which a manager will have some degree of control, as the first two splits at the top of a classification tree indicated (i) the extent of the infested area, (ii) reaction time and (iii) sanitary measures as being the most relevant factors for success (Fig. 3). The probability of eradication success was twice as high (66.7%) for infested areas below 4905 ha than for those above this threshold (32.5%). For infested areas smaller than 4905 ha, the probability for successful eradication was close to 90% if critical sanitary measures - such as banning the transfer of potentially contaminated material - were applied (Terminal Node 1). Even if no sanitary measures were applied, the probability of success for the small-scale infestation still exceeded 80% in man-made habitats (Terminal Node 2) as well as in (semi)-natural habitats invaded by species that escaped from cultivation (Terminal Node 3). The campaigns against species that invaded in (semi)-natural habitats via other pathways than escape from cultivation were much more likely to succeed in the Americas (59.3%; Terminal Node 4) than in Europe and Australasia (11.5%; Terminal Node 5). If the infested area was larger than 4905 ha, commencing the eradication campaign within 11 months of the problem first being noticed increased the chance of successful eradication threefold, compared to reacting after this period (46.6% vs. 15.2%). If the reaction was fast in this way, the probability of success was further doubled if the infestation source was identified by advanced diagnostic tools such as molecular methods (Terminal Node 6).

Factors that never appeared as important in the models included the pest type (agricultural, forestry, neither  =  conservation), the accessibility of the infested area, the proportion of infested habitat at the onset of the eradication campaign, control measures other than sanitary control, the frequency of introduction, pathways other than escape from cultivation, and also the level of self-declared coordination between involved parties.

## Discussion

### Location-specific Factors: The Role of Habitat, Geography, Spatial Scale and Pathway

Our results show that eradication success is not randomly distributed and that factors determining the outcome of eradication campaigns can be identified, with both location- and event-specific factors playing important roles, while species-specific characteristics were of minor importance. Across all analyses, eradications in man-made habitats (a location-specific factor) were more likely to succeed than campaigns carried out in (semi-)natural habitats. Man-made habitats included regularly or recently cultivated agricultural, horticultural and domestic habitats as well as constructed industrial and other artificial habitats. The reason why eradications in such habitats are more successful may lie in the fact that species invasions in agricultural and horticultural sectors are generally perceived as generating economic losses. Moreover, greenhouse crops are grown for economic reasons and usually have a large monetary value for the owner. It can therefore be assumed that owners have a strong vested interest in quickly eradicating the infestation in order to minimize economic losses and to prevent spread to other growers.

Outside greenhouses, the eradication campaigns in man-made habitats are generally more successful in Australasia. It can be hypothesized that as Australia, New Zealand and other regions in this part of the world are among those suffering particularly badly from biological invasions, organisms that are targeted for eradication represent such serious environmental and economic problems that they are carried out with extreme rigor. However, information on commitment and effort was not available to test this hypothesis directly. Furthermore, agricultural exports are especially important for both the New Zealand and the Australian economy and the “pest free status” for important agricultural pests is a valuable asset for exporters. Apart from Australasia, the success of eradication campaigns in man-made habitats depends on the spatial extent of invasion, being more successful at local and international than at regional and national scales. This seems to reflect that small infestations at the local scale are easier to eradicate, but might also imply that they are easier to monitor after the campaign so that reinvasions are detected earlier than at larger scales. The high likelihood of success at the international scale may be attributed to international coordination that is a crucial prerequisite to start such a large scale campaign, as it implies targeting species for control irrespective of national borders. It may also reflect the fact that international campaigns are carried out mainly against high-priority pests (i.e. the boll weevil *Anthonomus grandis*, plum pox virus (PPV) or brown rot *Ralstonia solanacearum*), which are likely to receive higher-than-average funding and commitment than smaller regional or national projects. Unfortunately, there was not enough information on funding available to directly test this hypothesis. For regional and national projects the success can be constrained if international cross-border collaboration is lacking. In seminatural habitats, plant species that were introduced on purpose (this result is specific to plants as other organisms analysed are not cultivated or kept in captivity) were more likely to be successfully eradicated than those introduced by other pathways. The ornamental pathway, with species introduced on purpose as a commodity sensu [44], delivers disproportionately more invasive plant species [45], [46], [47] due to preadaptation, care and time to generate sufficient propagule pressure, which is provided by humans through cultivation [48]. On the other hand, it has been shown recently that plant species introduced unintentionally become less frequently invasive but invade a wider range of seminatural habitats than species introduced in association with a commodity [49]. It may be that the biological traits of unintentionally introduced species that contribute to their invasion success in seminatural habitats are the same as those that provide them with increased resistance to eradication efforts.

The lower probability of the net effect of eradication success on islands than on mainlands (Fig. 2F) reflects the differences in data structure between both environments. The success on mainlands is predominantly determined by the interaction of introduction pathway with the type of habitat, with plants escaping from cultivation being easier to eradicate (and resulting in 83.3% success), while on islands it is determined by the feasibility of access to the infested areas (resulting in 65.5% success rate; analysis not shown). These complex interdependencies might explain why we found no difference in eradication success between island and mainland eradications in our previous paper [28].

### Manipulating Event-specific Factors: Increasing the Chance for Eradication Success

Event-specific factors affecting the outcome of eradication campaigns, such as the extent of the infested area, reaction time and measures of sanitary control can be taken into account and even be manipulated to some degree by authorities dealing with invasive species management. We previously found that the spatial extent of the outbreak is the only robust contingent factor significantly affecting the outcome of eradication campaigns against a wide range of organisms [28]. A study addressing weed eradications in California supports this assertion, by concluding that with a realistic (i.e. finite) amount of resources, the eradication of an invasive plant is unlikely to be successful beyond an infestation range size of 1000 ha [14]. Our analysis here further indicates that (i) initiating the campaign before the extent of infestation reaches a critical threshold, e.g. starting with eradication within four years of the problem being first noticed, (ii) paying special attention to plant species introduced as ornamentals, and (iii) applying sanitary measures can all substantially increase the probability of eradication success. It is also important to note that the role of residence time in a site, i.e. the time period over which a particular invasion has been running in the infested area, changes as the invasion progresses and only appears to have an important effect in relatively new invasions. If the action is taken within four years since the start of the invasion or its detection, eradication is likely; later, chances rapidly decrease. However, the effect of such a rapid response is no longer obvious when targeting invasions progressing for longer than 50 years. Another issue to be considered by management authorities when they collect background information prior to commencing an eradication campaign concerns the identification of the source of infestation. Provided the reaction is fast, the probability of success is doubled if modern diagnostic (i.e. molecular) methods are used to identify the source of infestation, probably reflecting a higher accuracy of correct species identification, compared to conventional methods. Therefore, potentially higher costs incurred by more expensive identification methods are likely to pay back in terms of achieving the goal.

### Management Implications: Need for Better Data

In this study, we could not analyze all factors considered to be important for eradication success, particularly socioeconomic factors. For example, the extent of stakeholder coordination, the degree of public support and the ‘quality’ of the project team personnel are all believed to be important for eradication success [12], but difficult to assess quantitatively for a large number of campaigns. In an attempt to estimate the level of stakeholder coordination, we asked contributors to assess such coordination, on a scale ranging from “no coordination”, “existing coordination” to “well functioning coordination”. Of the 148 replies, 120 considered the degree of coordination as “well functioning”, 27 as “existing” and only one as non-existing. Thus, apparently, the level of coordination is generally perceived high enough as not to limit eradication success. However, this self-assessment did not provide us with a sufficient range of different values needed to investigate the importance of coordination for eradication success, i.e. we did not have enough campaigns in which coordination was perceived as insufficient. This can mean two things: (1) either coordination in the practice of eradication campaigns is generally high and therefore no source of concern for managers, or (2) self-evaluation does not capture the “true” level of coordination and therefore that our analysis underestimates the influence of coordination on eradication success. Hence, a framework to properly assess and quantify socioeconomic factors such as “level of coordination”, “degree of public support”, or “quality and motivation of the team” is needed in order to compare their influence on eradication success. Currently data quantifying coordination or commitment are not recorded, and consequently they cannot be analyzed. This issue must be addressed in the future in collaboration with social scientists by establishing indicators for public support, stakeholder cooperation and the ‘quality’ of the eradication team. Even socioeconomic factors that are seemingly straightforward to quantify like eradication effort could not be analyzed because sufficiently detailed and reliable data on eradication costs and manpower used was too often lacking.

Several species-specific factors could also not be included in our cross-taxonomic analysis, because it is not apparent how even simple traits, for example, the body mass of distinct organisms like fungi, bacteria, viruses, insects or plants can be directly compared. We found only weak indications of taxonomic differences, indicating that species traits might not explain eradication success in general, but are probably more useful in analyses within taxa. Hence, the present study gives an insight into how factors that can be analyzed to date are relevant for eradication success.

To summarize, since a number of factors that seemed to be important for eradication success were not included we cannot postulate that the factors we identified as influencing eradications are the most important drivers in absolute terms. Accounting for these missing factors would undoubtedly further contribute to a better understanding as to which factors affect the success of eradication campaigns and control measures against invasive alien species in general. However, being based on a range of taxonomic groups, the factors identified as important in our study may be considered as reasonably general.

How can the results of this study thus be efficiently translated into practice, i.e. in terms of management recommendations? Besides the factors mentioned above that can be manipulated, one area where progress can be made relatively quickly relates to the importance of *a priori* information on the invading species. In pest risk-assessment schemes for screening potentially invasive species prior to their introduction, information on whether or not the species is invasive elsewhere is of crucial importance and of high predictive power [6], [50]–[53]. Similarly, quality information on the invasive organism targeted for eradication (i.e. preparedness) increases the likelihood of eradication success. This has been suggested before [15] but never tested in concert with other factors (except [28] where it was found to have no substantial effect). Although rapid response and available funds are crucial, understanding the biology of the species is equally important [15]. Therefore, attention should be given to fully document and report experiences of eradication campaigns and to make such reports publicly available, possibly in a global database. Such reports are often difficult to publish in scientific journals because of their descriptive character, which is often impossible to back-up with an appropriate experimental design with replications. But even if they do not meet standard scientific criteria, they do convey valuable information for pest managers. A global database of management/eradication campaigns would undoubtedly help pest managers worldwide to act rapidly and effectively in the event of an outbreak. A simple decision support system based on these findings would also be useful.

## Supporting Information

Table S1 List of eradication campaigns.
(XLS)

## References

[1] Pimentel D, McNair S, Janecka J, Wightman J, Simmonds C, et al (2001) Economic and environmental threats of alien plant, animal, and microbe invasions. Agr Ecosyst Environ 84: 1–20.

[2] Kettunen M, Genovesi P, Gollasch S, Pagad S, Starfinger U, et al.. (2009) Technical Support to EU Strategy on Invasive Alien Species (IAS) - Assessment of the Impacts of IAS in Europe and the EU. Brussels, Belgium: Institute for European Environmental Policy.

[3] Vilà M, Basnou C, Pyšek P, Josefsson M, Genovesi P, et al (2009) How well do we understand the impacts of alien species on ecosystem services? A pan-European, cross-taxa assessment. Front Ecol Environ 8: 135–144.

[4] Vilà M, Espinar JL, Hejda M, Hulme PE, Jarošík V, et al (2011) Ecological impacts of invasive alien plants: A meta-analysis of their effects on species, communities and ecosystems. Ecol Lett 14: 702–708. 21592274 10.1111/j.1461-0248.2011.01628.x

[5] Winter M, Schweiger O, Klotz S, Nentwig W, Andriopoulos P, et al (2009) Plant extinctions and introductions lead to phylogenetic and taxonomic homogenization of the European flora. Proc Natl Acad Sci USA 106: 21721–21725. 20007367 10.1073/pnas.0907088106 PMC2792159

[6] Nentwig W, Kühnel E, Bacher S (2010) A generic impact-scoring system applied to alien mammals in Europe. Cons Biol 24: 302–311. 10.1111/j.1523-1739.2009.01289.x 19604296

[7] Pyšek P, Jarošík V, Hulme PE, Pergl J, Hejda M, et al (2012) A global assessment of alien invasive plant impacts on resident species, communities and ecosystems: The interaction of impact measures, invading species’ traits and environment. Glob Change Biol 18: 1725–1737.

[8] Hulme PE (2006) Beyond control: Wider implications for the management of biological invasions. J Appl Ecol 43: 835–847.

[9] Baker RHA, Battisti A, Bremmer J, Kenis M, Mumford J, et al (2009) PRATIQUE: A research project to enhance pest risk analysis techniques in the European Union. EPPO/OEPP Bulletin 39: 87–93.

[10] Pyšek P, Richardson DM (2010) Invasive species, environmental change and management, and health. Annu Rev Environ Res 35: 25–55.

[11] FAO (2007) Glossary of Phytosanitary Terms (ISPM 5). Rome, Italy: FAO.

[12] Simberloff D (2009) We can eliminate invasions or live with them. Successful management projects. Biol Invas 11: 149–157.

[13] Myers JH, Savoie A, Randen E (1998) Eradication and pest management. Annu Rev Entomol 43: 471–491. 9444755 10.1146/annurev.ento.43.1.471

[14] Rejmánek M, Pitcairn MJ (2002) When is eradication of exotic pest plants a realistic goal? In: Veitch CR, Clout MN, eds. Turning the Tide: The Eradication of Invasive Species. Gland, Switzerland and Cambridge, UK: IUCN SSC Invasive Species Specialist Group. 249–253.

[15] Simberloff D (2003b) How much information on population biology is needed to manage introduced species? Cons Biol 17: 83–92.

[16] Simberloff D (2008) We can stop the invasion juggernaut! High- and low-tech success stories. Neobiota 7: 5–18.

[17] Bomford M, O’Brien P (1995) Eradication or control for vertebrate pests? Wildlife Soc Bull 23: 249–255.

[18] Myers JH, Simberloff D, Kuris AM, Carey JR (2000) Eradication revisited: Dealing with exotic species. Trends Ecol Evol 15: 316–320. 10884695 10.1016/s0169-5347(00)01914-5

[19] Clout MN, Veitch CR (2002) Turning the Tide of Biological Invasion: The potential for Eradicating Invasive Species. Gland, Switzerland and Cambridge, UK: IUCN SSC Invasive Species Specialist Group.

[20] Genovesi P (2007) Limits and potentialities of eradication as a tool for addressing biological invasions. In: Nentwig W, ed. Biological Invasions, Ecological Studies 193. Berlin & Heidelberg: Springer-Verlag. 385–402.

[21] Mack RN, Lonsdale WM (2002) Eradicating invasive plants – hard-won lessons from islands. In: Veitch CR, Clout M, eds. Turning the Tide: The Eradication of Invasive Species. Gland, Switzerland and Cambridge, UK: IUCN SSC Invasive Species Specialist Group. 164–172.

[22] Simberloff D (2003a) Eradication-preventing invasions at the outset. Weed Sci 51: 247–253.

[23] Courchamp F, Chapuis J, Pascal M (2003) Mammal invaders on islands: Impact, control and control impact. Biol Rev 78: 347–383. 14558589 10.1017/s1464793102006061

[24] Genovesi P (2005) Eradications of invasive alien species in Europe: A review. Biol Invas 7: 127–133.

[25] Brockerhoff EG, Liebhold AM, Richardson B, Suckling DM (2010) Eradication of invasive forest insects: Concepts, methods, costs and benefits. N Z J Forest Sci 40: S117–S135.

[26] Dahlsten DL, Garcia R (1989) Eradication of Exotic Pests: Analysis with Case Histories. New Haven, USA: Yale University Press.

[27] Sosnowski MR, Fletcher JD, Daly AM, Rodoni BC, Viljanen-Rollinson SLH (2009) Techniques for the treatment, removal and disposal of host material during programmes for plant pathogen eradication. Plant Pathol 58: 621–635.

[28] Pluess T, Cannon R, Jarošík V, Pergl J, Pyšek P, et al (2012) When are eradication campaigns successful? A test of common assumptions. Biol Invas 14: 1365–1378.

[29] Breiman L, Friedman JH, Olshen RA, Stone CJ (1984) Classification and Regression Trees. Belmont, California, USA: Wadsworth International Group.

[30] De’ath G, Fabricius KE (2000) Classification and regression trees: A powerful yet simple technique for ecological data analysis. Ecology 81: 3178–3192.

[31] Friedman JH (1999) Stochastic Gradient Boosting. Technical report. Stanford University, Dept. of Statistics.

[32] Friedman JH (2001) Greedy function approximation: A gradient boosting machine. Ann Stat 29: 1189–1232.

[33] Jarošík V (2011) CART and related methods. In: Simberloff D, Rejmánek M, eds. Encyclopedia of Biological Invasions. Berkeley and Los Angeles, USA: University of California Press. 104–108.

[34] Hochachka WM, Caruana R, Fink D, Munson A, Riedewald M, et al (2007) Data-mining discovery of pattern and process in ecological systems. J Wildlife Manag 71: 2427–2437.

[35] Steinberg G, Golovnya M (2006) CART 6.0 User’s Manual. San Diego, USA: Salford Systems.

[36] Hastie TJ, Tibshirani RJ, Friedman JH (2001) The Elements of Statistical Learning: Data Mining, Inference, and Prediction. New York: Springer.

[37] Blackburn TM, Duncan RP (2001a) Establishment patterns of exotic birds are constrained by non-random patterns in introduction. J Biogeogr 28: 927–939.

[38] Blackburn TM, Duncan RP (2001b) Determinants of establishment success in introduced birds. Nature 414: 195–197. 11700555 10.1038/35102557

[39] Davies CE, Moss D (2003) EUNIS Habitat Classification. Paris, France: European Topic Centre on Nature Protection and Biodiversity.

[40] Steinberg D, Colla P (1995) CART: Tree-structured Non-parametric Data Analysis. San Diego, USA: Salford Systems.

[41] Cutler DR, Edwards TC, Beard KH, Cutler A, Hess KT, et al (2007) Random forests for classification in ecology. Ecology 88: 2783–2792. 18051647 10.1890/07-0539.1

[42] Bourg NA, McShea WJ, Gill DE (2005) Putting a cart before the search: Successful habitat prediction for a rare forest herb. Ecology 86: 2793–2804.

[43] Breiman L, Cutler A (2004) Random Forests TM. An Implementation of Leo Breiman’s RFTM by Salford Systems. San Diego, USA: Salford Systems.

[44] Hulme PE, Bacher S, Kenis M, Klotz S, Kühn I, et al (2008) Grasping at the routes of biological invasions: A framework for integrating pathways into policy. J Appl Ecol 45: 403–414.

[45] Hanspach J, Kühn I, Pyšek P, Boos E, Klotz S (2008) Correlates of naturalization and occupancy of introduced ornamentals in Germany. Persp Plant Ecol Evol Syst 10: 241–250.

[46] Lambdon PW, Pyšek P, Basnou C, Hejda M, Arianoutsou M, et al (2008) Alien flora of Europe: Species diversity, temporal trends, geographical patterns and research needs. Preslia 80: 101–149.

[47] Liebhold AM, Brockerhoff EG, Garrett LJ, Parke JL, Britton KO (2012) Live plant imports: the major pathway for forest insect and pathogen invasions of the US. Front Ecol Environ 10: 135–143.

[48] Mack RN (2000) Cultivation fosters plant naturalization by reducing environmental stochasticity. Biol Invas 2: 111–122.

[49] Pyšek P, Jarošík V, Pergl J (2011) Alien plants introduced by different pathways differ in invasion success: Unintentional introductions as greater threat to natural areas? PLoS ONE 6: e24890. 21949778 10.1371/journal.pone.0024890 PMC3174229

[50] Pheloung PC, Williams PA, Halloy SR (1999) A weed risk assessment model for use as a biosecurity tool evaluating plant introductions. Journal of Environmental Management 57: 239–251.

[51] Křivánek M, Pyšek P (2006) Predicting invasions by woody species in a temperate zone: A test of three risk assessment schemes in the Czech Republic (Central Europe). Diversity Distrib 12: 319–327.

[52] Weber J, Panetta FD, Virtue J, Pheloung P (2009) An analysis of assessment outcomes from eight years’ operation of the Australian border weed risk assessment system. J Environ Manag 90: 798–807. 10.1016/j.jenvman.2008.01.012 18339471

[53] Leung B, Roura-Pascual N, Bacher S, Heikkilä J, Brotons L, et al.. (2012) TEASIng apart alien species risk assessments: a framework for best practices. Ecology Letters, doi: 10.1111/ele.12003. 10.1111/ele.12003 23020170

